# Molecularly Imprinted Sol-Gel-Based QCM Sensor Arrays for the Detection and Recognition of Volatile Aldehydes

**DOI:** 10.3390/s17020382

**Published:** 2017-02-16

**Authors:** Chuanjun Liu, Bartosz Wyszynski, Rui Yatabe, Kenshi Hayashi, Kiyoshi Toko

**Affiliations:** 1 Research and Development Center for Taste and Odor Sensing, Kyushu University, 744, Motooka, Nishi-ku, Fukuoka 819-0395, Japan; wyszynski.pawel.bartosz.939@m.kyushu-u.ac.jp (B.W.); yatabe@nbelab.ed.kyushu-u.ac.jp (R.Y.); hayashi@ed.kyushu-u.ac.jp (K.H.); toko@ed.kyushu-u.ac.jp (K.T.); 2 Graduate School of Information Science and Electrical Engineering, Kyushu University, 744, Motooka, Nishi-ku, Fukuoka 819-0395, Japan

**Keywords:** molecularly imprinted sol-gel, QCM sensor array, aldehyde biomarker, sensor array optimization

## Abstract

The detection and recognition of metabolically derived aldehydes, which have been identified as important products of oxidative stress and biomarkers of cancers; are considered as an effective approach for early cancer detection as well as health status monitoring. Quartz crystal microbalance (QCM) sensor arrays based on molecularly imprinted sol-gel (MISG) materials were developed in this work for highly sensitive detection and highly selective recognition of typical aldehyde vapors including hexanal (HAL); nonanal (NAL) and bezaldehyde (BAL). The MISGs were prepared by a sol-gel procedure using two matrix precursors: tetraethyl orthosilicate (TEOS) and tetrabutoxytitanium (TBOT). Aminopropyltriethoxysilane (APT); diethylaminopropyltrimethoxysilane (EAP) and trimethoxy-phenylsilane (TMP) were added as functional monomers to adjust the imprinting effect of the matrix. Hexanoic acid (HA); nonanoic acid (NA) and benzoic acid (BA) were used as psuedotemplates in view of their analogous structure to the target molecules as well as the strong hydrogen-bonding interaction with the matrix. Totally 13 types of MISGs with different components were prepared and coated on QCM electrodes by spin coating. Their sensing characters towards the three aldehyde vapors with different concentrations were investigated qualitatively. The results demonstrated that the response of individual sensors to each target strongly depended on the matrix precursors; functional monomers and template molecules. An optimization of the 13 MISG materials was carried out based on statistical analysis such as principle component analysis (PCA); multivariate analysis of covariance (MANCOVA) and hierarchical cluster analysis (HCA). The optimized sensor array consisting of five channels showed a high discrimination ability on the aldehyde vapors; which was confirmed by quantitative comparison with a randomly selected array. It was suggested that both the molecularly imprinting (MIP) effect and the matrix effect contributed to the sensitivity and selectivity of the optimized sensor array. The developed MISGs were expected to be promising materials for the detection and recognition of volatile aldehydes contained in exhaled breath or human body odor.

## 1. Introduction

The sensing of volatile aldehydes has attracted a continuously growing interest in various fields. It is well known that vapors of aldehydes such as formaldehyde, benzaldehye and nonanal, are typical indoor pollutants related to sick building syndrome [1]. Volatile hexanal and nonanal present in the exhaled breath are identified by many studies as biomarkers related to lung cancer [2,3,4,5]. In addition, as important metabolic intermediates of lipid peroxides, the increased level of nonanal reflects the oxidative stress or chronic airway inflammation [6,7,8,9,10,11]. Therefore, the detection of volatile aldehydes has been considered as an effective approach for the early detection of cancers as well as the monitoring of health status. It is, however, difficult to detect aldehydes selectively due to the complex composition of volatile organic compounds contained in exhaled breath. Moreover, the concentration of aldehydes in breath samples is as low as ppb level [3]. Most conventional gas sensors cannot meet the requirement of sensitivity and selectivity for the detection of aldehyde vapors. So far, only Masuda and Itoh et al. have reported SnO_2_-based nonanal sensors specifically aimed at the early detection of lung cancer [12,13]. By the combination of nanostructured SnO_2_ with noble metal catalysts, they realize a sub-ppm-level detection of the nonanal vapor. Although the sensitivity is improved greatly, their sensors show a lack of selectivity like most metal oxide semiconductor sensors. Therefore, the development of sensing materials with both high sensitivity and selectivity on volatile aldehydes is very important for the aldehyde-related biomarker sensors.

Molecularly imprinting polymers (MIPs) have been developed as an effective approach to prepare artificial receptors for specific molecular recognition and used in various sensing applications. The recognition ability derives from the specific cavities which fit the target molecules in size, shape and functional groups. MIPs receptors can be used in the recognition of not only large biomolecules, proteins and cells, but also volatile odorants or gas molecules [14,15,16,17,18,19,20]. Our previous works have demonstrated that the MIP technology is an effective approach for the detection and recognition of various odorant molecules [21,22,23,24,25]. The matrix of MIPs includes both organic polymers and sol-gel ceramic materials. Compared to polymer-based MIPs, the molecularly imprinted sol gels (MISGs) are characterized by: (1) mild preparation conditions; (2) various morphologies (bulk solids, fibers, films, etc.); (3) easily modified with organic groups; (4) high performance such as thermal stability, porous, rigid and optical properties [15,26,27]. Therefore, more and more attention has been paid to the gas/vapor sensing based on MISGs in recent years [14,15,16,27,28,29,30,31]. The aim of this work is to develop molecular recognition materials which can be used in the fabrication of both sensing devices and preconcentration devices on volatile aldehydes. Therefore, we focus our attention on inorganic MIPs in view of their high environmental and thermal stability.

A great variety of MISGs have been reported for chemical sensor applications [14,16,27]. Both silicon and titanium alkoxides are used as matrix precursors to fabricate MISGs [28,32,33,34,35,36,37,38,39]. Generally, the Si-based MISGs show advantages in the variety of molecular structures of silicon alkoxides while the Ti-based MISGs show advantages in high sensitivity and thermal stability. In addition, the addition of extra functional monomers has been found helpful for the increase in recognition ability of the matrix due to the increased imprinting effect during the sol-gel process as well as the increased interactions with targets during the sensing. In this study, MISGs with different matrix precursors, functional monomers and template molecules were developed for the detection and recognition of three typical aldehyde biomarker vapors: hexanal, nonanal and benzaldehyde. These MISGs were coated on quartz crystal microbalance (QCM) electrodes to fabricate multiple sensor arrays. The sensitivity and selectivity of the sensor array were quantitatively evaluated on the basis of various statistical analysis such as principle component analysis (PCA), multivariate analysis of variance (MANCOVA) and hierarchical cluster analysis (HCA). The prepared MISGs were optimized to fabricate a five-channel sensor array by which the highly sensitive detection and highly selective recognition on the three aldehydes were realized. It was suggested that both the matrix effect and the MIP effect contributed to the discrimination performance of the optimized sensor array. 

## 2. Materials and Methods

### 2.1. Materials

TEOS and TBOT used as the matrix precursors were purchased from Wako Pure Chemical Industries, Ltd., (Tokyo, Japan). Aminopropyltriethoxysilane (APT), and diethylaminopropyltrimethoxysilane (EAP) (Wako Pure Chemical Industries, Ltd.), trimethoxy-phenylsilane (TMP) (Sigma-Aldrich, Co. LLC, Japan, (Tokyo, Japan), were used as the functional monomers. Hexanoic acid (HA), nonanoic acid (NA) and benzoic acid (BA) used as the temples, titanium chloride (TiCl_4_) as a catalysis, isopropanol and ethanol as solvents, were also purchased from Wako Pure Chemical Industries Ltd. All of the reagents were used as received. In view of the instability of the aldehydes, the freshly purchased hexanal (HAL), nonanal (NAL) and bezaldehyde (BAL) were used to generate the vapors.

### 2.2. Synthesis of MISGs

The MISGs were prepared according to approaches reported by Lieberzeit et al. [29,33,35,36,39]. TiO_2_-based MISGs were prepared by dissolving 50 μL of template molecules in 2 mL of isopropanol. Then, 150 μL of TBOT and 50 μL of functional monomer were mixed into the solution. Afterwards, 25 μL of TiCl_4_ was added to initiate the hydrolysis and condensation. The reaction mixtures were heated in a water bath at 60 °C for 1 h while stirring. SiO_2_-based MISGs were prepared by dissolving 150 μL of TEOS, 50 μL of functional monomers, 50 μL of templates and 100 μL of H_2_O in 2 mL of ethanol. The reaction mixtures were stirred at 60 °C for 24 h. 

### 2.3. Coating of Sensing Layer

The sensing layers were prepared by spin coating of 5 μL of the above MISGs on QCM electrodes (two sides) with a speed of 3000 rpm. 9 MHz AT-cut quartz crystal electrodes embedded between vacuum-deposited Au (0.5 cm diameter) were used in this study. The MISGs-coated QCM electrodes were dried at 130 ℃ for 1h. This process has been reported enough to not only remove the templates from the layer materials but also produce smooth surface [36]. After that, the electrodes were then kept in vacuum at room temperature before the vapor sensing. 

### 2.4. QCM Measurement

The frequency change of the QCM electrodes were collected by a multiple channel QCM analyzer system (QCA 922, Seiko EG & G, Tokyo, Japan). The details of the system can be referred to our previous work [21,22]. By a standard gas generator (PD-1B-2, GASTEC Corporation, Kanagawa, Japan), volatile aldehyde vapors with different concentrations could be generated by using different types of the diffusion tube, temperatures of the chamber and flow rates of the diluted air. In this study, the D-30 diffusion tube was used and the chamber temperature was set as 50 ℃. For each vapor sample, three flow rates (0.3, 0.4 and 0.5 mL/min) were set, and for each flow rate the measurement was carried out with repeated three cycles (20 min air and 10 min vapor for each cycle). Therefore, totally 27 response datasets were obtained for the three aldehydes. The calculation of the precise concentrations of the vapors by measuring the diffusion coefficient failed due to the instability of aldehyde vapors in the air (autoxidation or degradation) [40]. A GC/MS analysis confirms the existence of corresponding carboxylic acid in the generated aldehyde vapors but the amount is found at a trace level (data not shown). It was estimated that the concentrations of the aldehyde vapors lied in the order of several to several tens of ppm by according to technical parameters of the standard gas generator as well as the reported references [12,13]. Blank samples obtained without putting any aldehyde liquids into the chamber were also tested since some MISGs showed response on the on/off switching possibly due to the moisture fluctuation of the flow path. The statistical analysis was performed using R software (R 3.3.1 GUI 1.68 Mavericks build) freely available from http://www.R-project.org (accessed on 30 May 2016). 

## 3. Results

Details of the MISGs-coated sensor channels are presented in Table 1. A total of 14 MISGs were investigated in this study. Among them, S0 is a Ti-based NIP blank prepared by TBOT without the addition of any functional monomers as well as templates. S1–S8 are Ti-based MISGs and S9–S13 are Si-based MISGs. It was found that the film-forming properties of the MISGs during the spin coating process was dependent upon the materials of matrix precursors, functional monomers and template molecules. For example, Si-based NIP samples were not investigated because no layer remained on the QCM electrodes after the spin coating, probably due to the high hydrophobicity of the TEOS sol-gels. This phenomenon was similarly observed for NIP materials with the addition of functional monomers such as TMP. On the contrary, good coating films were obtained for all template-added sol-gels which could be observed with naked eye or by the frequency change of the QCM electrodes before and after the spin coating. This result indicated that the template molecules might influence the sol-gel process as well as the affinity of the MISGs with the electrode surface. In addition, rapid precipitation was observed for the APT-added sol-gels in the absence of organic acid templates. This might be related to a high pH value induced by APT, which accelerated the hydrolysis and condensation process of the sol-gel reaction. The Si-based MISGs showed higher viscosity than the Ti-based MISGs, and thus the coating amount of the Si-MISG was generally higher than that of the Ti-MISGs.

**Figure 1:**
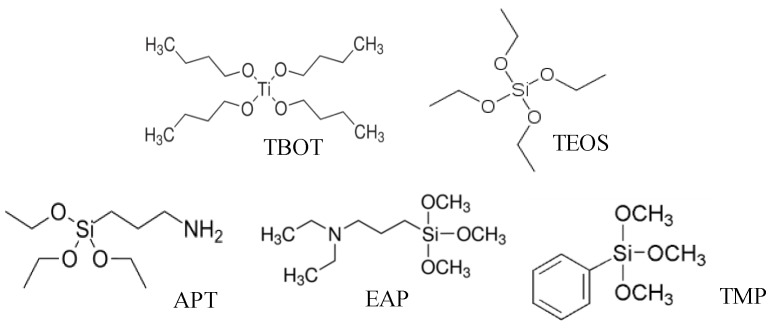
Molecular structure of two matrix precursors and three functional monomers.

**Figure 2:**
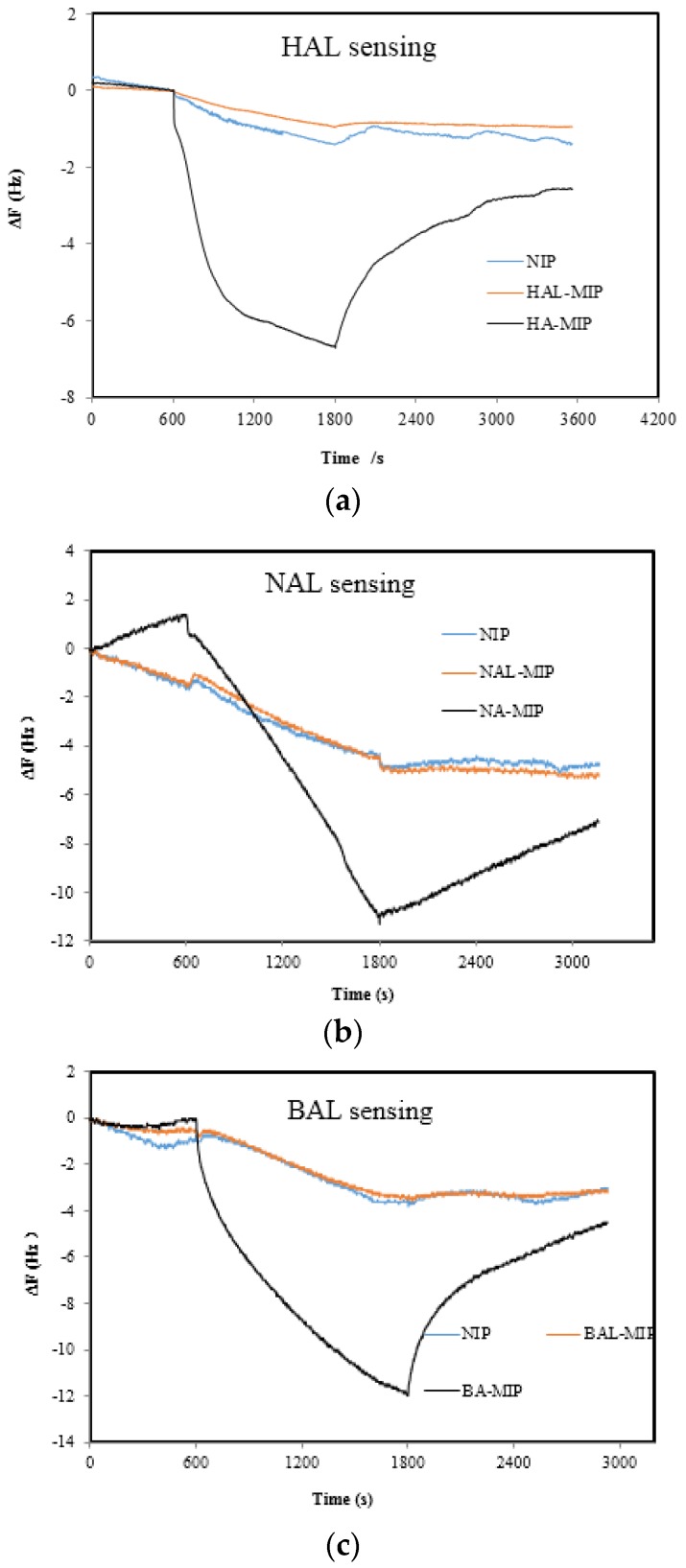
Response comparison of Ti-MISGs prepared by using aldehydes (Ald-) and corresponding organic acids (Acid-) as the templates: (**a**) hexanal and hexanoic acid; (**b**) nonanal and nonanoic acid; and (**c**) benzaldehyde and benzoic acid. The Ald-MIPs were prepared with the same conditions as S1, S2 and S3 except that the acid templates were replaced with aldehyde molecules.

The molecular structure of the used matrix precursors (TOES and TBOT) and functional monomers (APT, EPA and TMP) are shown in Figure 1. It is well known that strong interactions between the functional monomers and template molecules are good for the formation of three-dimensional binding pockest during the polymerization [14]. Many studies have confirmed the imprinting effect of both TEOS- and TBOT- based MISGs when organic acids are used as the template. This can be attributed to strong hydrogen bond interactions between the matrix and the organic acid molecules. Therefore, structurally analogous organic acid molecules instead of the aldehydes were used in this study in view of their stronger template effect in the formation of three-dimensional recognition cavities [21,22,41]. The use of structure analogs is a common approach in MIP researches to increase the imprinting effect. In a preliminary experiment, we compared the sensing character of TBOT-based MISGs by using aldehydes and their corresponding acid molecules as the templates. The results shown in Figure 2 confirm that the acid-templated MISGs demonstrate much higher sensitivity than the aldehyde-templated MISGs as well as the NISGs. In this study, we hoped that the addition of APT and EPA could further enhance the imprinting effect due to the introduction of amine groups. As for the addition of TMP, it was expected that the π-π interaction could be helpful for the cavity formation during the sol-gel process. At the same time, the π-π interaction also contributed to increase the recognition ability of the MISGs in the BAL sensing.

**Figure 3:**
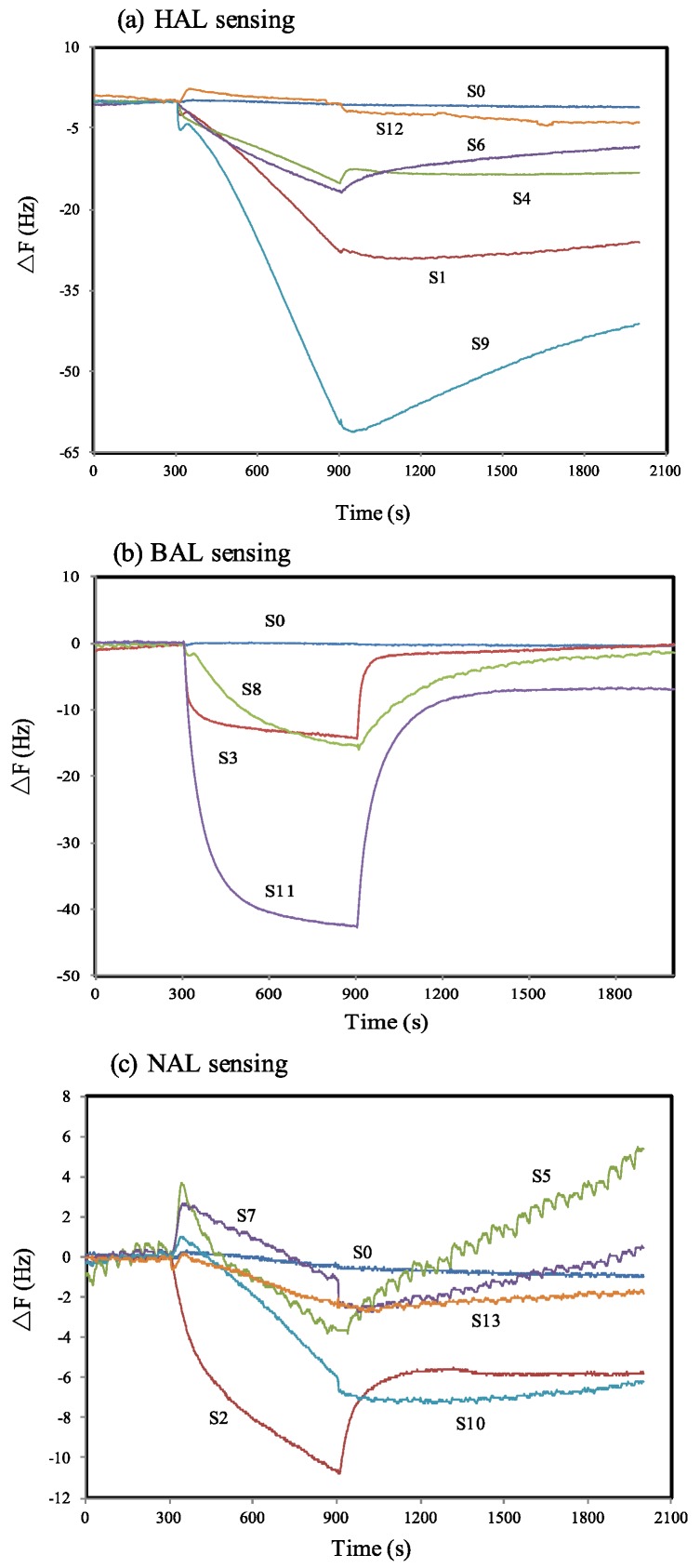
Response character of different MIP sensor channels on target vapors with a flow rate of 0.3 L/min: (**a**) HA-MIP on HAL, (**b**) BA-MIP on BAL and (**c**) NA-MIP on NAL.

Typical response characteristics of the QCM electrodes coated with the above MISGs to the three aldehydes are shown in Figure 3. It is noted that the non-imprinted sol-gel (S0) showed unobvious response to the three vapors. This may indicate that weak interactions between the sol-gel matrix and the targets. The responses of the MIP-coated electrodes varied widely depending on the materials used. For example, Figure 2a shows the responses of different HA-MIPs on the HAL vapor. Except the Si-APT channel (S12), other MIP channels showed obviously enhanced responses. Especially, the best response with a large frequency change of approximately 60 Hz was observed by the Si-based sol-gel added with EPA as the functional monomer (S9). In addition, it was found that the enhancement effect of APT and EPA on the Ti-based matrix (S4 and S6) was not apparent since their responses were less than that of the Ti-HA channel (S1). Figure 2b shows the responses of BA-MIPs on the BAL vapor. Compared with the Ti-BA channel (S3), the addition of TMP in the Ti-matrix (S8) showed no obvious effect. However, an outstanding effect was observed for the TMP-added Si-matrix (S11). Figure 2c shows the response of the NA-MIPs on the NAL vapor. Compared with the results of NAL and BAL, all responses were relatively smaller and the noise became apparent. We attributed it to the low vapor pressure and concentrations of the generated NAL vapors. Although the responses based on Ti-EPA (S7), Ti-APT (S5), Si-APT (S13) and Si-EPA (S10) were weak, a frequency change with values larger than 10 Hz was observed for S2, indicating a good imprinting effect of the Ti-NA MIP channel.

Although we hoped that the introduction of functional monomers into the sol-gel matrix could enhance the amount of target vapor molecules absorbed in the MISG layer, the results from Figure 2 demonstrated that the actual effect was influenced by many factors. Strong MIP effect was observed for the TEOS matrix, while it was not obvious for the TOBT matrix. The poor compatibility between the Si-based functional monomer and the Ti-matrix might be a possible reason for this. In addition, the hydrolysis and condensation rates of TiO_2_ sol-gels are generally faster than those of SiO_2_ sol-gels. It was possible that in the presence of functional monomers the sol-gel network might be insufficient for the Ti-based MISGs, and thus the MIP effect was not perfect if compared with the Si-based MISGs. In addition, compared with APT, EPA showed a much better enhancement effect on the target vapors, which was confirmed by the case of S9 (Si-EPA HA-MIP) on HAL and S10 (Si-EPA NA-MIP) on NAL. It is suggested that the secondary amine of EPA has much stronger electronegativity than that of the primary amine of APT, which led to a better imprinting effect during the sol-gel process as well as a stronger recognition ability of the MISGs in the vapor sensing. 

**Figure 4:**
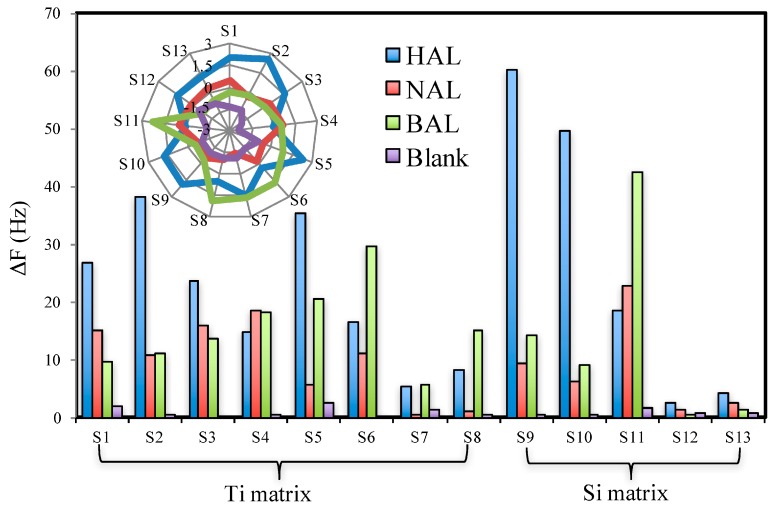
Response pattern of a 13 MISGs-coated sensor array on the three aldehydes and a blank. The vapors were generated with a flow rate of 0.3 L/min and the response of each channel was not normalized by the coating amount. Inset is a scaled radar chart of the 13 sensor channels.

The results shown in Figure 3 demonstrate that the highly sensitive detection of aldehyde vapors can be realized by rational design of MISGs. However, just high sensitivity is not enough for the application of the sensor in the early detection of cancers or the monitoring of health status, owing to the complexity of odorant compounds contained in exhaled breath as well as human body odor. In order to achieve this purpose, both the sensitivity and selectivity of the prepared MISGs are evaluated in the following step. The response pattern of a data set by using the 13 MISGs coated sensor array on the three aldehyde vapors is illustrated in Figure 4. Because the response of the channels to NAL is relatively small as shown in Figure 2c, the noise responses caused by the on/off flow switching were recorded together for a comparison. We called these samples as air blanks which means no diffusion tubes put in the sample chamber of the standard gas generator. The normalized pattern of the data set is illustrated by the radar chart of the inset in Figure 3. The datasets of the sensor array on vapors generated with different flow rates (27 datasets for the three aldehyde vapors and three datasets for the blank) were further analyzed by PCA (Figure 4). It can be seen that the target vapors can be clearly separated from each other from both the radar chart and the PCA score plot. The above results confirm the selectivity of the sensor array based on the prepared MISGs. 

**Figure 5:**
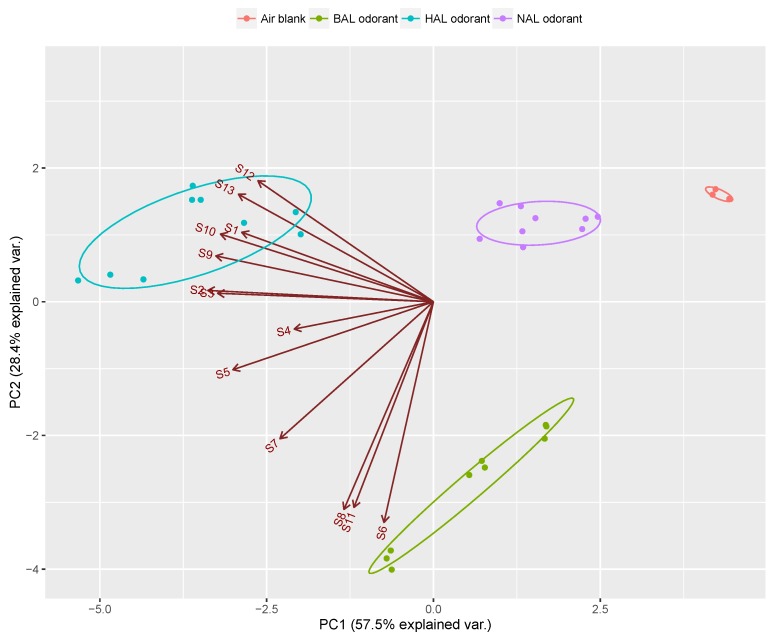
PCA map based on QCM sensor electrodes coated with 13 MISGs.

Although the multiple QCM analyzer is used in this work and the sensor channels can be added according to need, the results shown in Figure 3 demonstrate that not all channels are reversible and repeatable. In addition, it is expected that the discrimination of the target vapors can be achieved by a sensor array with the smallest possible number of channels. The loading analysis shown by the biplot of Figure 5 indicated that some channels had high correlation due to their similar loading vectors in magnitude and direction. In addition, some sensors showed relatively low loading values (such as S1 and S4), which indicated a low contribution of these channels to the total response of the array. These channels can be considered as redundant channels which will not only increase the complexity, cost and data processing time of the sensor system, but also degrade the performance of the sensor array [42]. Moreover, although the aldehyde vapors were discriminated successfully, the sensing mechanism of the MISGs is not clear and needs to be clarified. Therefore, the optimization of the prepared 13 MISGs was carried out in order to determine a sensor array with the best sensing performance as well as to understand the recognition mechanism.

Many methods have been reported and used in the optimization of sensor arrays [43,44,45,46,47,48,49,50,51]. The basic principle of the optimization is to maximize both the selectivity and the diversity of the sensor array. In this study, multivariate analysis of covariance (MANCOVA) was applied for evaluating the selectivity of individual sensor elements in the vapor discrimination. MANCOVA is a statistical method that is used to test differences among means of several groups with respect to more than one dependent variable. The discrimination ability of individual sensors with respect to the sample was evaluated by the F-value of Wilk’s Λ statistic. A large F-value means that the data groups are well separated. That is to say, the sensor channel holds high discrimination ability in the array. The F-values of 13 channels were calculated by MANCOVA and sorted in a descending order in Table 2. The channels of S7, S5, S4 and S1 showed very low F-values while the channels of S10, S9, S3 and S6 showed very high F-values. This result basically agrees with the loading analysis of individual channels shown in Figure 4. Therefore, the F-values can be used as a quantitative standard to evaluate the selectivity of the individual MISGs.

**Figure 6:**
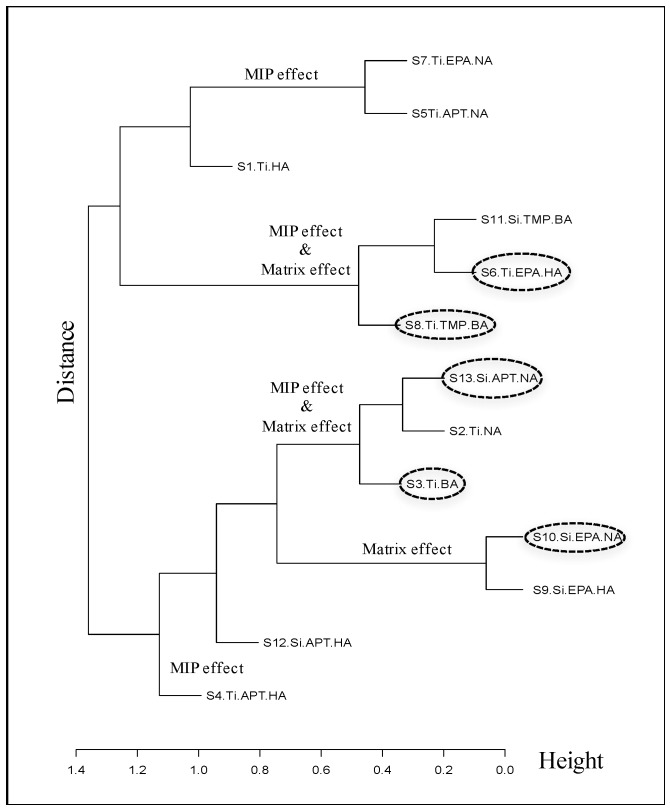
Hierarchical cluster dendrogram based on PC1-PC5 loading matrix of 13 sensors.

The diversity of the sensor array was evaluated by using cluster analysis. Cluster analysis is widely used to discriminate between response vectors in n-dimensional space by enhancing their difference. It is also used to find a set of clusters for which samples within a cluster are more similar than samples from different clusters [52]. Hierarchical clustering analysis (HCA) creates a hierarchy of clusters which is represented in a tree structure called dendrogram. The PCA results demonstrated that the proportion of variance of PC1 to PC5 was 57.5%, 28.4%, 7.7%, 2.5% and 1.8%, respectively. The accumulative proportion of PC1 and PC2 shown in Figure 4 was only 85.9%, while an accumulative proportion of 97.9% was obtained from PC1 to PC5. As a result, the HCA analysis was carried out by using the PAC loading matrix of PC1 to PC5. The hierarchical cluster dendrogram based on Ward’s method is shown in Figure 6. According to the clustering result, a high degree of similarity was observed for S5/S7, S6/S11, S2/S13 and S9/S10. We can choose either of them to fabricate a sensor array without redundant channels. In addition, HCA also gave us a hint to understand the recognition mechanism by analyzing the material character of the clustered MISGs. For example, S7 and S5 were same in the template molecule NA but different in the matrix. This may indicate that the discrimination ability of this cluster (S5 and S7) was based on the MIP effect (the same effect can be seen in the case of S4/S12). On the contrary, the cluster consisting of S9 and S10 held the same matrix (Si-EPA) but the different templates (NA and HA), which may mean that the discrimination ability of this cluster was based on the matrix effect. For other clusters such as S1/S6/S8 and S2/S3/S13, the situation became complicated and both MIP and matric effect might contribute to the discrimination ability of these MISGs.

**Figure 7:**
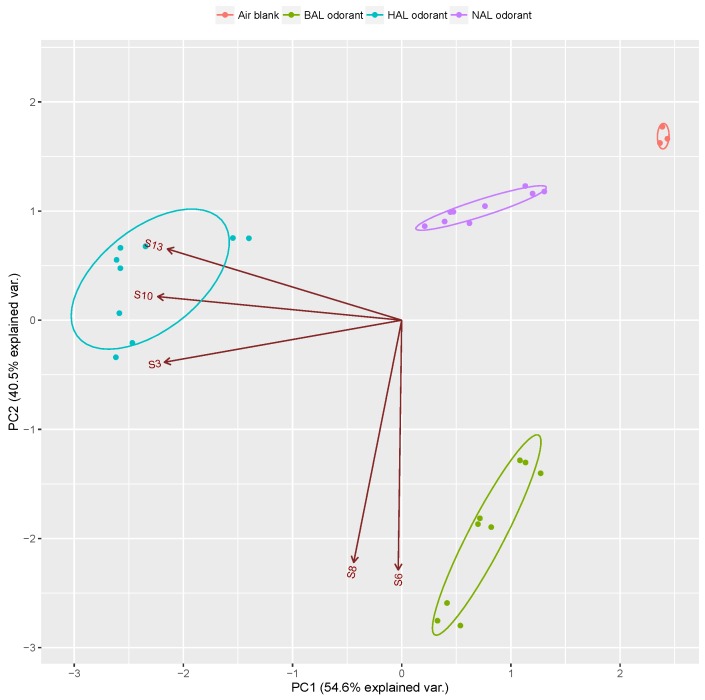
PCA map based on the optimized sensor array.

According to the criteria that the F-values should be high and that the clusters should not overlap in the clusters [42], the 13 channel sensor array was optimized to a five channel array including S3, S6, S8, S10 and S13. The optimized sensor array consists of a HA-MIP (Ti-EPA matrix), two BA-MIPs (Ti- and Ti-TMP matrix) and two NA-MIPs (Si-APT and Si-EPA). Results of PCA analysis based on the dataset of the optimized sensor array is shown in Figure 7.

**Figure 8:**
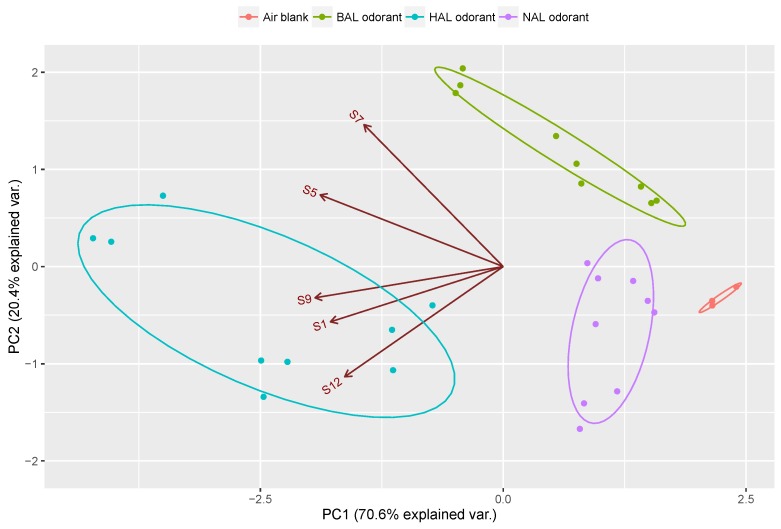
PCA map based on a randomly selected sensor array.

It can be seen that the three aldehyde vapors were well discriminated in the PC1-PC2 space with a cumulative proportion of 95.1%, which was higher than the result shown Figure 4. In addition, the observed confidence interval (95% confidence level) of the optimized array was much smaller than that of the 13-channel array, which from another side verified a higher recognition accuracy of the optimized array than the original array. In order to further confirm the optimization effect, a sensor array consisting of five randomly selected channel from the 13 MISGs pool was fabricated and the sensitivity and selectivity of the two arrays were quantitatively compared. Figure 8 shows the PCA result of the random sensor array consisting of S1, S5, S7, S9 and S12. The sensitivity and selectivity of the two sensor arrays were quantified by using a method proposed by Chaudry et al. [43]. According to their method, the sensitivity of a sensor array is defined as the sum of root sum (SRSS), and the selectivity is defined as sum of Euclidean distance (SED). Briefly, a measurement of the overall sensitivity of any one-sensor element can be calculated by root sum square (RSS) of the normalized response for given analyte. Therefore, the sensitivity for an array of sensor (SRSS) can be calculated by summing the RSS values for the selected sensors. The selectivity is simply a measurement of how different the two or more response vectors are from one another, which is quantified by calculating the Euclidean distance between pairs of the response matrix. The calculated results shown in Table 3 demonstrate that although the SRSS vales of the two sensors were almost same, the optimized array showed a higher SED (14.87) value than that of the random array (10.77).

**Figure 9:**
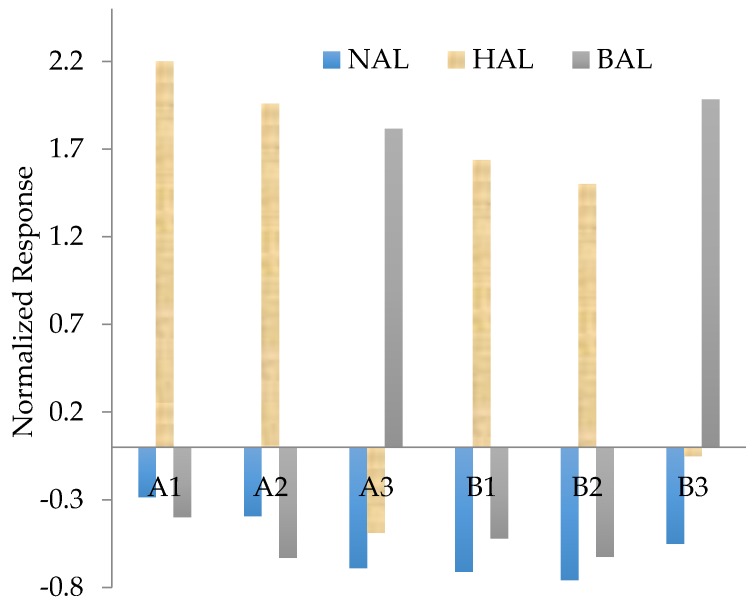
Normalized response pattern of sensor channels.

We noticed that the Si-based MIP with the addition of EPA showed both high sensitivity and selectivity on HAL and NAL if compared with MISGs added with other functional monomers. This result might indicate that the Si-EPA-MISG materials was particularly suitable for aliphatic aldehyde sensing. It is well known that the imprinting effect of MIP materials is influenced by the preparation condition such as the ratio between the functional groups, the templates and the matrix. In the above experiment, the ratio of the matrix to functional monomer were all fixed as 150 μL/50 μL to prepared the MISGs. Then, we adjusted the ratio of TEOS to EPA/TMP to 100 μL/100 μL, and investigated the influence of the increase in the functional monomer on the selectivity of sensor array. As indicated in Table 4, the sensor array A represents the new prepared MISGs with the ratio of TESO to EPA/TMP of 100/100, while sensor array B represents the original MISGs with the ratio of 150/50. For both arrays, the amount of template molecules was the same as 50 μL. The normalized response of the 6 channels on three aldehyde vapors are shown in Figure 9. Because the response patterns of the array A (A1, A2, A3) and the array B (B1, B2, B3) were visually similar, the values of SRSS and SED were calculated to quantify the performance of the two arrays. As shown in Table 5, the array A shows both higher SRSS and SED values than the array B. Moreover, it was found that a mixed sensor array C (B1, A2, A3) showed an obviously different pattern from the array A and B. The calculated SED value of the array C confirmed its higher sensitivity than the other two. These results demonstrated that the performance of sensor array could be further improved by tuning the composition of the MISGs. In this study, only three vapors were targeted. The kinds of alkoxides (both the matrix precursors and the functional monomers) that were investigated were also limited. However, the results reported in this study reveals that it is possible to detect and discriminate more vapors or their mixtures by using diversified matrix precursors, functional monomers and template molecules.

## 4. Conclusions

QCM sensor arrays based on molecularly imprinted sol-gel (MISG) materials were investigated in the present study for the detection and recognition of volatile aldehyde vapors. The MISGs were prepared by using different matrix precursors, functional monomers and template molecules. Data analysis based on PCA, MANCOVA and HCA were applied to select the MISGs and to optimize the sensor array. The detection and recognition of three aldehyde vapors (hexanal, nonanal and benzaldehyde) with low concentrations were realized based on an optimized sensor array consisting of five MISGs. It was suggested that both the MIP and matrix effect made contribution to the discrimination ability of the sensor array. The approaches reported in this work, including both the MISG design and statistical analysis, can be used in systematic screening for molecular recognition materials. In view of their high sensitivity and selectivity on aldehyde vapors, the MISGs might be promising materials in the fabrication of sensor devices and preconcentration devices, and thus applied in the disease early detection as well as health status monitoring.

## Figures and Tables

**Table 1:** Detailed information of 14 sensor channels fabricated by molecularly imprinted sol gels (MISGs).

Sensor Number	Sensor Name Abbreviation	Matrix Materials	Functional Monomers	Template Molecules
S0	Ti-Blank (NIP)	TBOT	-	-
S1	Ti-HA-MIP	TBOT	-	HA
S2	Ti-NA-MIP	TBOT	-	NA
S3	Ti-BA-MIP	TBOT	-	BA
S4	Ti-APT-HA-MIP	TBOT	APT	HA
S5	Ti-APT-NA-MIP	TBOT	APT	NA
S6	Ti-EPA-HA-MIP	TBOT	EPA	HA
S7	Ti-EPA-NA-MIP	TBOT	EPA	NA
S8	Ti-TMP-BA-MIP	TBOT	TMP	BA
S9	Si-EPA-HA-MIP	TEOS	EPA	HA
S10	Si-EPA-NA-MIP	TEOS	EPA	NA
S11	Si-TMP-BA-MIP	TEOS	TMP	BA
S12	Si-APT-HA-MIP	TEOS	APT	HA
S13	Si-APT-NA-MIP	TEOS	APT	NA

**Table 2:** F values calculated by MANCOVA for 13 sensor channels.

Sensor	F-Value	Pr	MISG
S10	350.50	2.20 × 10^−16^	Si-EPA-NA-MIP
S9	283.69	2.20 × 10^−16^	Si-EPA-HA-MIP
S6	104.36	1.28 × 10^−14^	Ti-EPA-HA-MIP
S3	87.04	1.11 × 10^−13^	Ti-BA-MIP
S8	63.41	4.34 × 10^−12^	Ti-TMP-BA-MIP
S2	62.78	4.86 × 10^−12^	Ti-NA-MIP
S13	61.69	5.92 × 10^−12^	Si-APT-NA-MIP
S11	42.96	3.23 × 10^−10^	Si-TMP-BA-MIP
S12	31.07	9.43 × 10^−9^	Si-APT-HA-MIP
S1	24.29	1.04 × 10^−7^	Ti-HA-MIP
S4	22.80	1.89 × 10^−7^	Ti-APT-HA-MIP
S5	15.79	4.76 × 10^−6^	Ti-APT-NA-MIP
S7	11.57	5.29 × 10^−5^	Ti-EPA-NA-MIP

**Table 3:** Sensitivity (SRSS) and selectivity (SED) of the optimized and random sensor arrays.

	Sensor Number	SRSS	SED
Optimized array	3,6,8,10,13	3.35	14.87
Random array	1,5,7,9,12	3.36	10.77

**Table 4:** Sensor channels with different volume ratio of TEOS/EPA: A = 100/100 and B = 150/50.

Sensor Name	Matrix Materials	Functional Monomers	Template Molecules
A1	TEOS 100	EPA 100	HA50
A2	TEOS 100	EPA 100	NA50
A3	TEOS100	TMP 100	BA50
B1	TEOS 150	EPA 50	HA50
B2	TEOS 150	EPA 50	NA50
B3	TEOS 150	TMP 50	BA50

**Table 5:** SRSS and SED comparison of sensor arrays consisting of different channels.

Sensor Array	SRSS	SED
(A) A1, A2, A3	2.34	5.07
(B) B1, B2, B3	2.15	4.77
(C) B1, A2, A3	2.32	5.13
